# Cancer Risk After Radioactive Iodine Treatment for Hyperthyroidism: A Systematic Review and Meta-analysis

**DOI:** 10.1001/jamanetworkopen.2021.25072

**Published:** 2021-09-17

**Authors:** Sung Ryul Shim, Cari M. Kitahara, Eun Shil Cha, Seong-Jang Kim, Ye Jin Bang, Won Jin Lee

**Affiliations:** 1 Department of Preventive Medicine, Korea University College of Medicine, Seoul, Republic of Korea; 2 Radiation Epidemiology Branch, Division of Cancer Epidemiology and Genetics, National Cancer Institute, National Institutes of Health, Bethesda, Maryland; 3 Department of Nuclear Medicine, Pusan National University Yangsan Hospital, Yangsan, Republic of Korea; 4 Department of Nuclear Medicine, Pusan National University College of Medicine, Yangsan, Republic of Korea; 5 BioMedical Research Institute for Convergence of Biomedical Science and Technology, Pusan National University Yangsan Hospital, Yangsan, Republic of Korea

## Abstract

**Importance:**

Whether radioactive iodine (RAI) therapy for hyperthyroidism can increase cancer risk remains a controversial issue in medicine and public health.

**Objectives:**

To examine site-specific cancer incidence and mortality and to evaluate the radiation dose-response association after RAI treatment for hyperthyroidism.

**Data Sources:**

The Medline and Cochrane Library electronic databases, using the Medical Subject Headings terms and text keywords, and Embase, using Emtree, were screened up to October 2020.

**Study Selection:**

Study inclusion criteria were as follows: (1) inclusion of patients treated for hyperthyroidism with RAI and followed up until cancer diagnosis or death, (2) inclusion of at least 1 comparison group composed of individuals unexposed to RAI treatment (eg, the general population or patients treated for hyperthyroidism with thyroidectomy or antithyroid drugs) or those exposed to different administered doses of RAI, and (3) inclusion of effect size measures (ie, standardized incidence ratio [SIR], standardized mortality ratio [SMR], hazard ratio [HR], or risk ratio [RR]).

**Data Extraction and Synthesis:**

Two independent investigators extracted data according to the Meta-analysis of Observational Studies in Epidemiology (MOOSE) guidelines. Overall quality assessment followed the recommendations of United Nations Scientific Committee on the Effects of Atomic Radiation. The SIR and SMRs and the RRs and HRs were pooled using random-effects meta-analysis.

**Main Outcomes and Measures:**

Cancer incidence and mortality for exposure vs nonexposure to RAI therapy and by level of RAI administered activity.

**Results:**

Based on data from 12 studies including 479 452 participants, the overall pooled cancer incidence ratio was 1.02 (95% CI, 0.95-1.09) and the pooled cancer mortality ratio was 0.98 (95% CI, 0.92-1.04) for exposure vs nonexposure to RAI therapy. No statistically significant elevations in risk were observed for specific cancers except thyroid cancer incidence (SIR, 1.86; 95% CI, 1.19-2.92) and mortality (SMR, 2.22; 95% CI, 1.37-3.59). However, inability to control for confounding by indication and other sources of bias were important limitations of studies comparing RAI exposure with nonexposure. In dose-response analysis, RAI was significantly associated with breast and solid cancer mortality (breast cancer mortality, per 370 MBq: 1.35; *P* = .03; solid cancer mortality, per 370 MBq: 1.14; *P* = .01), based on 2 studies.

**Conclusions and Relevance:**

In this meta-analysis, the overall pooled cancer risk after exposure to RAI therapy vs nonexposure was not significant, whereas a linear dose-response association between RAI therapy and solid cancer mortality was observed. These findings suggest that radiation-induced cancer risks following RAI therapy for hyperthyroidism are small and, in observational studies, may only be detectable at higher levels of administered dose.

## Key Points

**Question:**

Is radioactive iodine (RAI) therapy for hyperthyroidism associated with an increased cancer risk?

**Findings:**

This systematic review and meta-analysis, including 12 observational studies and 479 452 participants, found that RAI therapy was not associated with a significant increase in overall cancer risk or site-specific cancer incidence or mortality, except for thyroid cancer. However, a linear radiation dose-response association between RAI therapy and solid cancer mortality was observed, based on 2 studies with information on RAI administered dose.

**Meaning:**

Considering the global use of RAI therapy for hyperthyroidism, further studies are needed to provide quantitative estimates of site-specific cancer risks associated with RAI therapy, particularly at higher doses.

## Teaser

This systematic review and meta-analysis examines site-specific cancer incidence and mortality and evaluates the radiation dose-response association after radioactive iodine treatment for hyperthyroidism.

## Introduction

Hyperthyroidism, a form of thyrotoxicosis, is a clinical state characterized by inappropriately high tissue thyroid hormone levels.^1^ The prevalence of hyperthyroidism ranges from 0.1% to 2.9% worldwide^2^ and is approximately 1.2% (0.5% overt and 0.7% subclinical) in the United States. The most common causes include Graves disease, toxic multinodular goiter, and toxic adenoma.^3^

Radioactive iodine (RAI) has been used to treat hyperthyroidism for more than 7 decades. According to the American Thyroid Association guidelines, RAI therapy has been strongly recommended with moderate-quality evidence as 1 of the 3 major treatments (ie, RAI, antithyroid drugs, and thyroidectomy) for patients with overt Graves hyperthyroidism. RAI treatment is preferred in situations in which greater value is placed on the definitive control of hyperthyroidism, the avoidance of surgery, and the potential adverse effects of antithyroid drugs, and a lower value is placed on the need for lifelong thyroid hormone replacement and the rapid resolution of hyperthyroidism.^1^ The European Thyroid Association guidelines also recommend RAI therapy to be considered for patients who prefer this approach.^4^

However, the extensive use of RAI therapy has raised concerns regarding its potential carcinogenic and leukemogenic effects. RAI therapy for thyroid cancer^5,6,7^ and for hyperthyroidism^8,9^ has been associated with an increased risk of subsequent malignant neoplasms, whereas other studies^10,11^ have reported no associations. However, a recent study by Kitahara et al,^12^ showing a dose-response association between RAI treatment and the risk of cancer death, has challenged the notion that RAI therapy for hyperthyroidism does not have long-term adverse effects.

Previously, a systematic review and meta-analysis reported the cancer risks after RAI exposure for hyperthyroidism.^13^ Several studies for RAI therapy for hyperthyroidism and subsequent cancer risk were published thereafter.^14,15,16^ Most, but not all, of these studies were summarized in a 2020 narrative review^17^ describing the association between RAI therapy for hyperthyroidism and cancer risk. However, to our knowledge, there have been no systematic reviews or meta-analyses summarizing the dose-response association of RAI therapy for hyperthyroidism with subsequent cancer risk. In addition, a new quality assessment tool for radiation epidemiology studies was recommended by the United Nations Scientific Committee on the Effects of Atomic Radiation (UNSCEAR),^18^ but it has yet to be applied in a systemic review of RAI treatment studies. Therefore, an updated systematic review of the evidence on cancer risks following RAI therapy for hyperthyroidism is warranted.

The current study is a systematic review and meta-analysis of the published literature on RAI treatment for hyperthyroidism and site-specific cancer incidence and mortality using a new quality assessment tool for radiation epidemiology studies from UNSCEAR. In this study, we estimated the overall effect sizes of cancer incidence and mortality by site for patients exposed to RAI therapy vs those unexposed. When possible, we evaluated the shape and magnitude of the dose-response association for RAI therapy and risk of these outcomes.

## Methods

This systematic review and meta-analysis was performed according to the standard Preferred Reporting Items for Systematic Reviews and Meta-Analyses (PRISMA) statement^19^ and the Meta-analysis of Observational Studies in Epidemiology (MOOSE) reporting guideline.^20^ Our protocol was registered in the PROSPERO International prospective register of systematic reviews database (CRD42020161142) prior to the study.

### Literature Search

The electronic databases Medline and Cochrane Library were screened up to October 2020 using the Medical Subject Headings terms and text keywords. The subject headings and text keywords included those related to population (eg, *hyperthyroidism*), interventions (eg, *iodine*), and outcomes (eg, *neoplasm*) (eTable 1 in the Supplement). The search terms were grouped according to the Boolean operators (ie, AND, OR, NOT). The searches were limited to human studies and were performed for all languages and study types. The same search strategy was adopted for Embase using Embase subject headings (Emtree). Additional studies were identified by 2 independent investigators (S.R.S. and E.S.C.) through manually searching conference abstracts, clinical trial databases, and reference lists.

### Study Selection

Study inclusion criteria were as follows: (1) the study population comprised patients who were diagnosed with primary cancer after RAI treatment for benign thyroid diseases, such as thyrotoxicosis and/or hyperthyroidism; (2) the interventions included the administration of RAI treatment; (3) the comparisons were specified for nonexposed iodine treatment groups, such as the general population and/or patients with hyperthyroidism receiving other treatment modalities (thyroidectomy or antithyroid drugs), or patients exposed to different administered doses of RAI; and (4) the outcomes were standardized incidence ratio (SIR), standardized mortality ratio (SMR), hazard ratio (HR), or risk ratio (RR) for incidence ratio and mortality after RAI treatment. For overlapping studies from the same cohort, the latest and most appropriate outcomes were selected by the consensus of all the investigators. Two investigators (S.R.S. and E.S.C.) independently screened the titles and abstracts of all the articles using the predefined inclusion criteria. The full-text articles were examined independently by all investigators to determine whether they met the inclusion criteria. Furthermore, the same authors independently extracted data using a data extraction form. The final inclusion of each article was determined by all investigators’ evaluation discussions. References and data for each included study were carefully cross-checked to ensure that no overlapping data were present and to maintain the integrity of the meta-analysis.

### Data Extraction

Information on the number of patients, mean age of patients, the proportion of female patients, country of study, treatment period, follow-up period, publication year, types of control groups, dose of RAI, effect size estimates, and adjusted covariates were extracted from the included articles, using a predefined data extract form. Because none of the studies included estimates of the organ dose of RAI except for the study by Kitahara et al,^16^ we used the mean total administered activity of individual studies. We then divided the patients into 3 dose groups (<309, 309-504, and ≥505 MBq) by the first and third quartiles to ensure reasonable visibility in all investigators’ assessment discussions.

### Quality Assessment

The overall quality assessment followed the recommendation of UNSCEAR 2017.^18^ We assessed the following 8 parameters: study participants, exposure, outcomes, design-specific bias, confounder control, statistical methods, reporting bias, and conflicts of interest. We graded each signaling question, risk-of-bias judgment, and overall quality assessment (eTable 2 and eFigure 3 in the Supplement).

### Statistical Analysis

SIR and SMR were used for external comparison analysis, and the RR and HR was used for internal comparison analysis. The RR and HR were used for dose-response meta-analysis (DRMA). The random-effects model published by DerSimonian and Laird^21^ was used to obtain the pooled overall incidence and mortality ratios with 95% CIs for outcomes. The statistical heterogeneity was evaluated using Cochran *Q* test and the *I*^2^ statistic. A metaregression analysis was conducted for each moderator. To examine potential moderators, we used a restricted maximum likelihood estimator of the variance of the true effects.^22^ Subgroup analyses were performed by number of patients, dose level, sex proportion, country, treatment period, follow-up time, control group, study quality, and effect size measures to test the stability and robustness of the results.

In reports regarding dose-response–associated cancer risks after RAI treatment, we conducted 2-stage DRMAs that comprised obtaining the regression coefficients of individual studies in the first stage and calculating the total coefficient by converging the weighted means of the regression coefficients of individual studies in the second stage.^23^ We also represented the linear and nonlinear dose-response associations for RAI and cancer risks graphically.^24^

The funnel plot illustrates the publication bias using standard error as the measure of study size and ratio measures of treatment effect. In addition, we conducted the Begg and Mazumdar rank correlation test^25^ and the Egger linear regression method test^26^ for publication bias. A 2-sided *P* ≤ .05 was considered significant. The analyses were conducted using R version 3.6.0 (R Foundation for Statistical Computing).

## Results

### Study Selection

**Figure 1.**
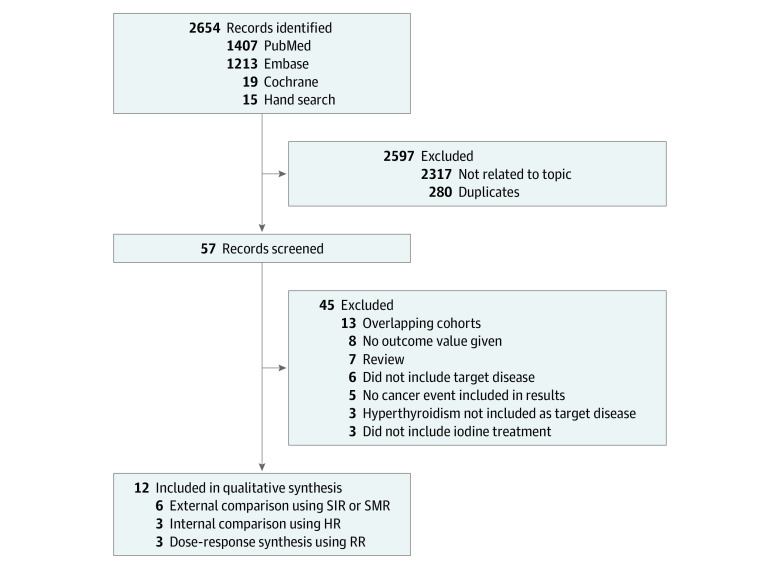
Flow Diagram of Study Selection Process HR indicates hazard ratio; RR, risk ratio; SIR, standardized incidence ratio; and SMR, standardized mortality ratio.

The initial search identified a total of 2639 articles from electronic databases (PubMed, 1407; Cochrane, 19; Embase, 1213). Of these, 2317 studies that contained data unrelated to the topic and overlapping data or appeared in more than 1 database were excluded. After a more detailed review, an additional 13 papers that were review studies or concerned nontarget diseases were eliminated. After screening the titles and abstracts, 44 studies were determined to be eligible for intensive screening. Of these, 32 studies were further excluded for the following reasons: no cancer events as results, 5 studies; no hyperthyroidism as the target disease, 3 studies; no iodine treatment, 3 studies; no effect estimates, 8 studies; and duplicate results in the same cohort, 13 studies (Figure 1). The data from the 2019 study by Kitahara et al^12^ was replaced by the data from the 2020 study by Kitahara et al,^16^ as the earlier publication was the only study assessing cancer risks by estimates of organ and/or tissue absorbed doses. Finally, 12 studies^8,9,10,11,14,15,16,27,28,29,30,31^ met our selection criteria for qualitative assessment, among which 9 pairwise meta-analyses (6 external comparisons^8,9,10,11,16,28^ using SIR or SMR and 3 internal comparisons^14,15,31^ using HR or RR) and 3 DRMAs^9,16,30^ were included in the quantitative meta-analysis. Two diagnostic studies^27,29^ were used for the qualitative assessment.

A systematic review of the 12 studies was conducted to assess the experimental differences and subject descriptions in detail (Table 1). Most of the studies were conducted in North America and Europe, with the main cohorts being the Cooperative Thyrotoxicosis Therapy Follow-up Study cohort (TTFUS)^10,16,28^ and Swedish Cancer Register.^8,9,14^ The range of the calendar years of treatment and mean per-patient follow-up duration in the quantitative meta-analysis were from 1946 to 2015 and 7.27 to 27 years, respectively, whereas the mean RAI therapeutic administered activity ranged from 259 to 507 MBq. Nine cohort studies^8,9,10,14,16,27,28,29,30^ were classified as high and moderate quality, and 3 studies^11,15,31^ were rated at relatively low and very low quality (eTable 2 in the Supplement).

**Table 1.** Characteristics of Cohort Studies on Cancer Risks After Radioactive Iodine Treatment for Hyperthyroidism

Source	Country; population	Sample size, No.^a^	Sex, No. (%)	Mean age	Control	Adjusted covariates	Treatment period	Mean follow-up, y	Lag time	Dose, MBq	Reporting outcomes	Reporting cancer site^b^
Goldman et al,^28^ 1988^c^	1 US hospital in TTFUS; hyperthyroidism	1762; 607 receiving RAI only; 799, RAI and other; 356, no RAI	Female, 1762 (100)	NA	US or Massachusetts standard population	Age, calendar time, sex, race-specific (White), region (Connecticut)	1946-1964	17.2	1 y	375.5	SIR	All, digestive organs, pancreas, breast, brain
Holm et al,^8^ 1991^d^	Sweden; hyperthyroidism	10 207	Female, 115 561 (83.1); male, 23 457 (16.9)	Male: 56; female: 57	Swedish Cancer Register	Age, sex, calendar year, region, dose	1950-1975	15	1 and 10 y	506	SIR	All, oral cavity, salivary glands, stomach, liver, pancreas, colon, rectum, lung, breast, female genital organs, male genital organs, kidney, bladder, brain, thyroid gland, parathyroid gland, lymphoma, multiple myeloma, leukemia, Hodgkin disease, non-Hodgkin disease
Hall et al,^9^ 1992^d^	Sweden; hyperthyroidism	10 552 (93% for hyperthyroidism; 7% for nonspecified thyroid disease)	Female: 126 523 (83.0); male: 25 883 (17.0)	Male: 56; female: 57	Swedish Cancer Register	Age, sex, calendar year, region, dose	1950-1975	15	1 and 10 y	507	SMR	All, digestive organ, respiratory tract, breast, female genital organs, male genital organs, kidney, bladder, nervous system, thyroid gland, lymphoma, leukemia
Ron et al,^10^ 1998^c^	TTFUS; hyperthyroidism	35 593; 8054, RAI only; 20 949, RAI and other; 10 876, surgery with or without drugs; 1177, drugs only	Female: 28 248 (79.4); male: 7345 (20.6)	46 at cohort entry	US standard population	Age, sex, race, calendar year, type of hyperthyroidism, time since treatment, dose	1946-1964	21	1 to >10 y	385	SMR	All, buccal cavity, digestive organ, esophagus, stomach, colorectal, liver, pancreas, larynx, lung, breast, uterus, ovary, prostate, bladder, kidney, thyroid gland, brain, myeloma, leukemia, CLL, non-CLL, lymphoma
Franklyn et al,^11^ 1999	UK regional cancer register; hyperthyroidism	7417 receiving RAI	Female: 6189 (83.4); male: 1228 (16.6)	56.6	UK Regional Cancer Register	Age, sex, calendar year, period	Cohort, 1950-1991; control group, 1971-1991	9.7	NA	307.7	SIR, SMR	All, lip-oral cavity and pharynx, digestive organ, stomach, pancreas, small bowel, respiratory and intrathoracic organs, breast, genitourinary organs, bladder, brain, thyroid gland, lymphatic and hemopoietic, lymphomas, leukemia
Hahn et al,^29^ 2001^e^	German Democratic Republic’s cancer registry; children examined for suspected thyroid disease	789 receiving RAI; 1118 with no exposure	Female: 584 (74.0); male: 205 (26.0)	≤18	German Democratic Republic cancer registry	Age, sex	Cohort, 1958-1978; nonexposure group, 1959-1986	20	NA	0.9	SIR	Thyroid gland
Dickman et al,^27^ 2003^e^	Sweden; patients receiving RAI for diagnostic purposes	24 010 with no prior exposure to external radiotherapy	Female: 18 488 (77.0); Male: 5522 (23.0)	≤75	Swedish Cancer Register	Age, sex, calendar year, region, dose	1950-1975	27	2 to >20 y	1.6	SIR	Thyroid gland
Metso et al,^30^ 2007^f^	Finland; hyperthyroidism	1399 receiving RAI; 1465, thyroidectomy	Female: 2336 (83.6); male: 457 (16.4)	62	Thyroidectomy, Finland nationwide Hospital Discharge Registry	Age, sex, treatment type, etiology of hyperthyroidism	1965-2002	9 in RAI group; 9.4, thyroidectomy group	3 mo	305	RR for mortality	All
Ryodi et al,^31^ 2015	Finland; hyperthyroidism	1814 receiving RAI; 4334, thyroidectomy	RAI: female, 1485 (81.9); male, 329 (18.1); surgery: female, 3719 (85.8); male, 615 (14.2)	59 in RAI group; 46 in thyroidectomy group	Thyroidectomy, Finland nationwide Hospital Discharge Registry	Age, sex, treatment type, etiology of hyperthyroidism	1986-2007	10	3 mo	259	HR for incidence and mortality	All
Giesecke et al,^14^ 2018	Swedish health care register (hyperthyroidism)	10 250 receiving RAI; 742, thyroidectomy	RAI: female, 8668 (84.6); male, 1577 (15.4); surgery: female, 633 (85.3); male, 109 (14.7)	65.1 in RAI group; 44.1 in thyroidectomy group	Thyroidectomy, Swedish Cancer Register	Age, sex, treatment period, comorbidities	1976-2000	16.3-22.3	NA	NA	HR of mortality	All
Gronich et al,^15^ 2020	Israel Clalit Health service register; hyperthyroidism	2829 receiving RAI; 13 808, ATD (thionamide)	Female: 12 304 (74.0); Male: 4333 (26.0)	51.9	ATD group	Age, sex, smoking history, BMI, Clalit district, socioeconomic status, diabetes, hypertension, use of aspirin and statins, adherence to mammography	2002-2015	7.27	1 y	685	HR for incidence	All, breast, colorectal, lung, thyroid, prostate, uterus and cervix, urinary, central nervous system, kidney, stomach pancreas, ovary, melanoma, non-Hodgkin lymphoma, leukemia, liver and bile ducts, head and neck, bone connective tissue, esophagus
Kitahara et al,^16^ 2020	TTFUS; hyperthyroidism	31 363; 7474 receiving RAI only; 12 115, RAI and other; 800, surgery only; 1138, drugs only; 9817 drugs and surgery	Female, 28 248 (79.4); male, 7345 (20.6); from Ron et al^10^	46 at cohort entry	US standard population	Age, sex, birth cohort, other risk factors, dose	1946-1964	26	5 y	397	SMR	All, breast, oral cavity, esophagus, stomach, colon, rectum, liver, pancreas, lung or bronchus, bladder, kidney, brain or central nervous system, thyroid gland, uterus, ovary, prostate

Abbreviations: ATD, antithyroid drug; BMI, body mass index; CLL, chronic lymphatic leukemia; HR, hazard ratio; NA, not available; RAI, radioactive iodine; RR, risk ratio; SIR, standardized incidence ratio; SMR, standardized mortality ratio; TTFUS, the Cooperative Thyrotoxicosis Therapy Follow-up Study.

^a^ The total sample size may not equal the numbers of the original articles because some patients were excluded from the analysis.

^b^ Publications where all malignant neoplasms were shown in individual studies are indicated by all.

^c^ TTFUS was a cooperative thyrotoxicosis therapy follow-up cohort study conducted at 25 US and 1 UK hospitals. In the TTFUS study, solid cancers were extracted from the 2002 study by Kitahara et al^16^ and blood cancers from the study by Ron et al.^10^ The data from the 2019 study by Kitahara et al^12^ have been replaced by the latest data from the 2020 study by Kitahara et al.^16^

^d^ These studies were conducted at a Swedish cohort of 7 hospitals.

^e^ Diagnostic study, used only for qualitative assessments.

^f^ Used only for the dose-response meta-analysis.

### Outcome Findings

Detailed findings of the cancer risks compared with control groups in the different body sites after RAI treatment for hyperthyroidism are described in Table 2 and eFigure 1 in the Supplement. The pooled overall incidence ratio in the meta-analysis was 1.02 (95% CI, 0.95-1.09). Cochran *Q* test showed moderate heterogeneity (*I*^2^ = 63.0%), and only the incidence of thyroid gland cancer was statistically significant at an incidence ratio of 1.86 (95% CI, 1.19-2.92). Furthermore, the pooled overall mortality ratio in the meta-analysis was 0.98 (95% CI, 0.92-1.04). Cochran *Q* test showed moderate heterogeneity (*I*^2^ = 57.2%). The association for mortality from thyroid cancer was statistically significantly higher (mortality ratio, 2.22; 95% CI, 1.37-3.59). In the subgroup analysis of external comparisons using SIR or SMR (incidence ratio, 1.01; 95% CI, 0.93-1.09; mortality ratio, 0.97; 95% CI, 0.91-1.04) and internal comparison using HR or RR (incidence ratio, 1.05; 95% CI, 0.92-1.19; mortality ratio, 1.05; 95% CI, 0.86-1.27), the summary effect size estimates did not reach statistical significance. Forest plots by cancer site are presented in eFigure 4 and eFigure 5 in the Supplement.

**Table 2.** Meta-analysis of the Incidence and Mortality Ratios of Cancer Risks After Radioactive Iodine Treatment for Hyperthyroidism

Cancer site^a^	Incidence ratio^b^				Mortality ratio^c^			
	Effect sizes, No.^d^	Heterogeneity^e^		Effect size (95% CI)	Effect sizes, No.^d^	Heterogeneity^e^		Effect size (95% CI)
		*I*^2^, %	*P* value			*I*^2^, %	*P* value	
**Internal comparison using HR**								
All	2	0	.88	1.02 (0.90-1.16)	2	0	.96	1.05 (0.86-1.27)
Digestive organ	5	0	.46	1.32 (0.90-1.95)	0	NA	NA	NA
Eye, brain, and other parts of the central nervous system	2	62.0	.10	0.68 (0.12-3.89)	0	NA	NA	NA
Female genital organs	2	0	.46	0.90 (0.48-1.69)	0	NA	NA	NA
Urinary tract	2	70.1	.07	0.80 (0.20-3.30)	0	NA	NA	NA
Lymphoid, hematopoietic, and related tissue	2	45.8	.17	1.43 (0.42-4.85)	0	NA	NA	NA
Overall estimates of internal comparison	20	8.8	.35	1.05 (0.92-1.19)	2	0	.96	1.05 (0.86-1.27)
**External comparison using SIR or SMR**								
All	3	93.2	<.001	0.91 (0.74-1.12)	2	91.9	<.001	0.99 (0.82-1.20)
Digestive organ	6	22.9	.26	1.03 (0.93-1.14)	8	44.0	.09	1.05 (0.94-1.18)
Eye, brain, and other parts of the central nervous system	3	43.4	.17	1.11 (0.58-2.14)	3	0	.58	0.97 (0.70-1.35)
Lip, oral cavity, and pharynx	3	0	.47	0.89 (0.61-1.31)	2	0	.32	0.72 (0.47-1.13)
Respiratory and intrathoracic organs	2	96.0	<.001	0.89 (0.41-1.93)	3	88.2	<.001	0.93 (0.69-1.24)
Breast	3	0	.90	1.04 (0.94-1.14)	3	0	.76	0.88 (0.77-1.00)
Genital organs								
Female	0	NA	NA	NA	3	82.1	<.001	0.96 (0.68-1.36)
Male	0	NA	NA	NA	2	80.0	.03	0.81 (0.48-1.34)
Urinary tract	3	86.5	<.001	1.05 (0.72-1.53)	5	0	.47	0.91 (0.78-1.06)
Thyroid gland	3	59.2	.09	1.86 (1.19-2.92)	3	0	.80	2.22 (1.37-3.56)
Lymphoid, hematopoietic, and related tissue	4	29.9	.23	0.81 (0.64-1.02)	7	0	.89	0.97 (0.80-1.16)
Overall estimates of external comparison	32	73.5	<.001	1.01 (0.93-1.09)	41	59.1	<.001	0.97 (0.91-1.04)
Overall estimates	52	63	<.001	1.02 (0.95-1.09)	43	57.2	<.001	0.98 (0.92-1.04)

Abbreviations: HR, hazard ratio; NA, not applicable; SIR, standardized incidence ratio; SMR, standardized mortality ratio.

^a^ All indicates publications in which all malignant neoplasms were shown; digestive organs, stomach, liver, pancreas, intestine, colon, and rectum; eye, brain, and other parts of the central nervous system, brain and nervous system; lip, oral cavity, and pharynx, salivary glands; respiratory and intrathoracic organs, lungs; male genital organs, prostate; urinary tract, kidney and bladder; and lymphoid, hematopoietic, and related tissues, lymphoma, multiple myeloma, and leukemia.

^b^ Incidence ratio calculated from SIRs in Goldman et al,^28^ Holm et al,^8^ and Franklyn et al^11^ and HRs in Ryodi et al and ^31^ Gronich et al.^15^

^c^ Mortality ratio calculated from SMRs in Hall et al,^9^ Ron et al,^10^ Franklyn et al,^11^ and Kitahara et al^16^ and HRs for mortality in Giesecke et al^14^ and Ryodi et al.^31^

^d^ Based on at least 2 effect sizes for each organ; the total number of effect sizes does not equal the sum of individual numbers due to overlap.

^e^ *P* value of Cochrane *Q* statistics for heterogeneity; some values below the decimal point differ according to the formula for calculating the standard error.

### Effect Size Modifiers

Table 3 provides a subgroup analysis and an overview of the metaregression analysis results. The metaregression analysis found that RAI administered activity dose, number of patients, follow-up period, and study quality were significantly associated with cancer risk. Particularly, the administered dose of RAI was significantly positively associated with cancer incidence among patients with hyperthyroidism (<309 MBq: effect size, 0.86 [95% CI, 0.74-1.01]; 309-504 MBq: effect size, 0.86 [95% CI, 0.68-1.09]; ≥505 MBq: effect size, 1.09 [95% CI, 1.03-1.16]; regression coefficient, 1.13 [95% CI, 1.06-1.21]; *P* < .001) and mortality (<309 MBq: effect size, 0.90 [95% CI, 0.82-1.00]; 309-504 MBq: effect size, 0.92 [95% CI, 0.84-1.01]; ≥505 MBq: effect size, 1.10 [95% CI, 1.02-1.18]; regression coefficient, 1.11 [95% CI, 1.04-1.19]; *P* = .002). There was no statistically significant difference between results using an external comparison (SIR and SMR) and those based on an internal comparison (HR and RR).

**Table 3.** Associations of Moderators With Overall Cancer Risk After Radioactive Iodine Treatment for Hyperthyroidism

Variables	Incidence ratio			Mortality ratio		
	Effect sizes, No.	Effect size (95% CI)	*P* value^a^	Effect sizes, No.	Effect size (95% CI)	*P* value^a^
No. of total patients						
≥10 000	38	1.09 (1.03-1.16)	<.001	13	1.09 (1.02-1.17)	<.001
<10 000	14	0.87 (0.76-0.99)		30	0.91 (0.85-0.97)	
Dose, MBq^b^						
<309	10	0.86 (0.74-1.01)	<.001	10	0.90 (0.82-1.00)	.002
309-504	4	0.86 (0.68-1.09)		20	0.92 (0.84-1.01)	
≥505	38	1.09 (1.03-1.16)		12	1.10 (1.02-1.18)	
Coefficient^c^	NA	1.13 (1.06-1.21)		NA	1.11 (1.04-1.19)	
Sex proportion						
≥80% of female	33	1.01 (0.94-1.09)	.82	23	1.00 (0.92-1.08)	.16
<80% of female	19	1.05 (0.88-1.25)		20	0.92 (0.84-1.01)	
Country						
United States	4	0.86 (0.68-1.09)	.39	20	0.92 (0.84-1.01)	.16
Others	48	1.02 (0.95-1.10)		23	1.00 (0.92-1.08)	
Treatment period						
Before 1980	32	1.01 (0.93-1.09)	.81	41	0.97 (0.91-1.04)	.57
After 1980	20	1.05 (0.92-1.19)		2	1.04 (0.86-1.27)	
Follow-up time, y						
≥10	24	1.08 (1.02-1.14)	.01	34	1.00 (0.93-1.07)	.14
<10	28	0.92 (0.80-1.04)		9	0.89 (0.89-0.99)	
Control group						
General population	32	1.01 (0.93-1.09)	.81	41	0.97 (0.91-1.04)	.60
Surgery and ATD	20	1.05 (0.92-1.19)		2	1.04 (0.86-1.27)	
Quality assessment^d^						
High and moderate	38	1.09 (1.03-1.16)	<.001	33	1.00 (0.93-1.07)	.21
Low	14	0.87 (0.76-0.99)		10	0.91 (0.82-1.00)	
Internal vs external comparison						
Standardized ratio	32	1.01 (0.93-1.09)	.81	41	0.97 (0.91-1.04)	.60
Hazard ratio	20	1.05 (0.92-1.19)		2	1.04 (0.86-1.27)	

Abbreviations: ATD, antithyroid drugs; NA, not applicable.

^a^ *P* value from metaregression analysis using the restricted maximum likelihood.

^b^ Divided at the first and third quartile.

^c^ Exponential regression coefficient.

^d^ Quality assessment follows the recommendations of the United Nations Scientific Committee on the Effects of Atomic Radiation.^18^

### Dose-Response Meta-analysis

**Figure 2.**
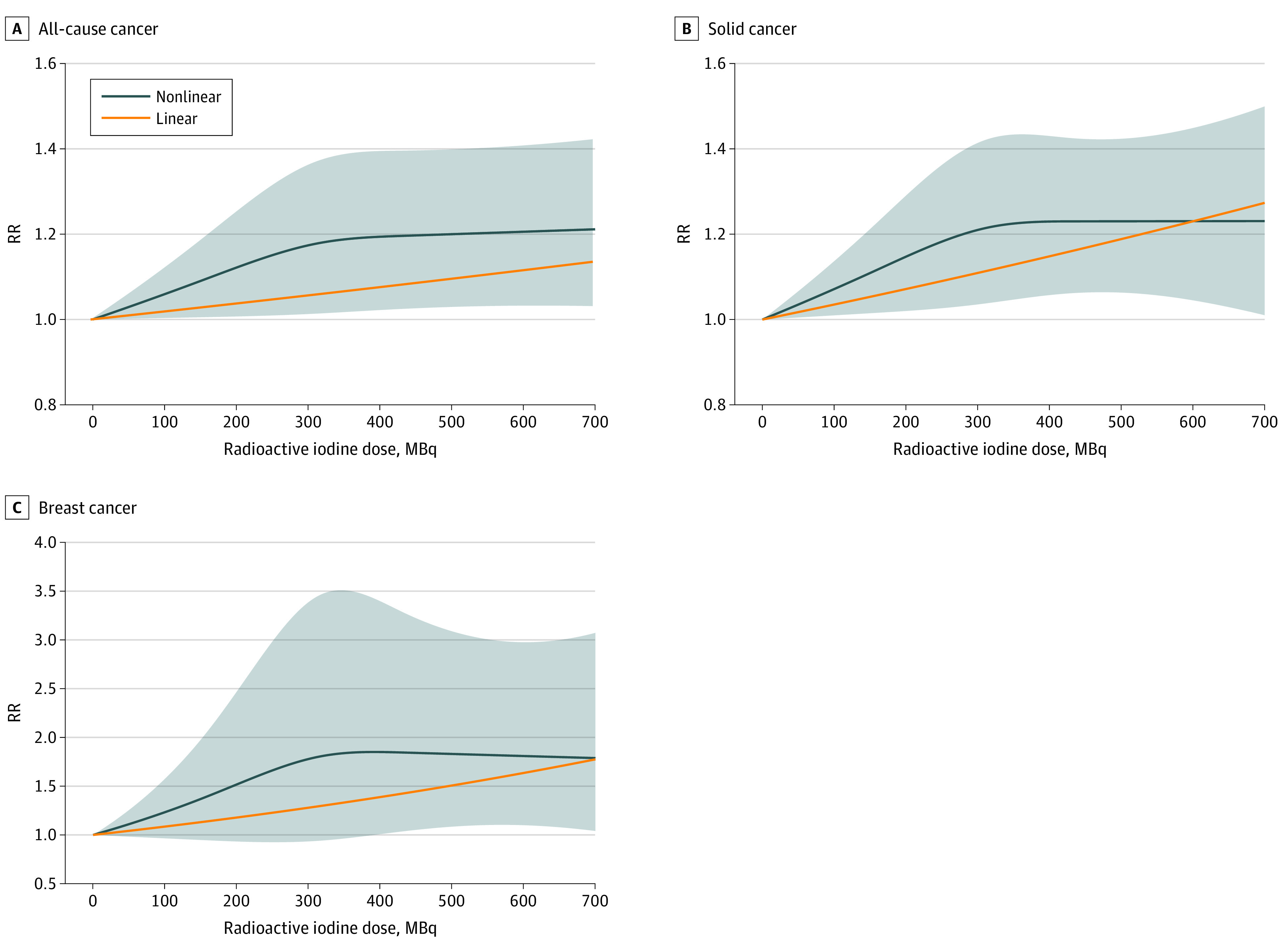
Dose-Response Associations Using Linear and Nonlinear Models for Radioactive Iodine Dose and Cancer Mortality Restricted cubic spline model used for nonlinear analysis. Shaded area indicates 95% CI of nonlinear model. A, All-cause cancer risk ratio (RR) calculated from studies by Hall et al^9^ and Metso et al.^30^ B, Solid cancer RR calculated from studies by Hall et al^9^ and Kitahara et al.^16^ The solid cancer RR reported by Hall et al^9^ synthesized the types of solid cancers, such as stomach, breast, kidney, and others, because the study did not report the whole solid cancer RR of the dose groups. C, Breast cancer RR calculated from studies by Hall et al^9^ and Kitahara et al.^16^

Figure 2 displays the linear and nonlinear associations for RAI dose-response mortality cancer risks of all-cause, solid, and breast cancers. Breast and solid cancer mortality increased with higher administered doses (breast cancer mortality, per 370 MBq: 1.35; *P* = .03; solid cancer mortality, per 370 MBq: 1.14; *P* = .01). The risk tended to flatten between 300 and 400 MBq in the nonlinear model.

### Publication Bias

Publication bias was evaluated in eFigure 2 in the Supplement. The funnel plots of the incidence and mortality ratios appeared symmetrical. The *P* values of the incidence and mortality ratios for Begg and Mazumdar correlation test and Egger regression coefficient test suggested that there was no evidence of publication bias or small-study effect in this meta-analysis.

## Discussion

This systematic review and meta-analysis found no significant risks of total cancer incidence or mortality after RAI therapy for hyperthyroidism, except for thyroid cancer. However, the DRMA found that increases in the RAI dose were associated with increases in mortality from solid cancer and breast cancer.

The risk of thyroid cancer mortality was elevated more than 2-fold (SMR, 2.22; 95% CI, 1.37-3.59) after RAI therapy for hyperthyroidism. All 3 included studies^9,11,16^ reported that elevated thyroid cancer mortality was associated with RAI therapy, and Ron et al^10^ reported that the SMR of thyroid cancer was 3.94 (95% CI, 2.52-5.86). One of possible reason may high dose of radiation exposure on thyroid gland. According to Kitahara et al^12^ the mean organ dose estimate for the thyroid gland in the TTFUS cohort study was 130 Gy, which is a substantially larger dose than those administered to other organs and tissues. High doses of radiation can cause DNA damage, which could be mediated via a combination of direct effects, through breakage of molecular bonds, or indirectly through the formation of free radicals, which lead to decreased thyroid function and/or thyroid size.^4^ However, Ron et al^10^ and Kitahara et al^12^ found no evidence of a dose-response association when modeling administered activity or absorbed dose to the thyroid gland.

The underlying conditions of the thyroid gland could be another possible reason for the increased risk of malignant thyroid tumor after RAI for hyperthyroidism. Thyroid-stimulating hormone and thyroid-stimulating antibodies, present in Graves disease, may play a role in carcinogenesis and tumoral growth, and hyperthyroidism is associated with a high incidence of thyroid carcinoma.^32,33^ Furthermore, tumors arising from hyperthyroid tissue show aggressive behavior.^34,35^ Therefore, we assumed that the present meta-analysis included patients who had hyperthyroidism as the target disease, implying that they already had risk of abnormal thyroid gland function.

Regarding the dose-response cancer risk after RAI therapy for hyperthyroidism, the present metaregression analysis found that overall cancer mortality among the study groups was significant. In DRMAs with studies reporting dose-response–associated cancer risks, the association with breast cancer mortality was stronger (1.35 per 370 MBq), while the association of solid cancer mortality was weaker (1.14 per 370 MBq) than that reported by Kitahara et al,^16^ but both associations were statistically significant and in the positive direction. The present meta-analysis similarly found an increasing cancer risk after RAI therapy for hyperthyroidism with higher administered dose. The studies that reported dose-response cancer risks by RAI doses were relatively higher quality than those of other included studies.

Although the methodological quality of the included studies was of an acceptable level (9 studies with high and moderate quality; 3 with low and very low quality), the quality of the evidence was rated as moderate at best. Most articles had a moderate or serious risk of bias with regard to exposure owing to lack of organ dose estimation, except for the study by Ron et al.^10^ However, most articles showed a low risk of bias with regard to study participants, outcomes, design-specific bias, and reporting bias due to included national cohort studies. Therefore, the ascertainment of RAI treatment for hyperthyroidism is unlikely to have caused a substantial bias in our findings.

### Limitations

This study has several limitations. First, the individual studies included patients who were not randomized to the treatments they received, and such observational studies may be vulnerable to confounding bias.^36^ However, most studies were evaluated as having relatively reliable quality for confounder control, although the individual studies may have been influenced by inadequate control for confounding by indication, which occurs when the reasons underlying the selection of a treatment are independently associated with risk of the outcome. Second, the number of studies included in the quantitative synthesis was relatively small for pooling risks of relatively uncommon cancer types despite the large sizes of the cohorts, and only 3 studies provided information of dose-response associations. In addition, the available data did not allow determination of cancer risk according to the etiology and severity of hyperthyroidism, cancer case ascertainment, and changes in treatment strategies over the past few decades. Third, we used the mean total administered activity of individual studies due to the lack of information on the organ or tissue absorbed dose of the RAI except for the study by Ron et al^10^ and Kitahara et al.^16^ However, risk estimates based on organ doses should be more reliable because organ dose estimates account for clinical parameters, including thyroid uptake and gland size; nonetheless, administered dose may be a reasonable proxy for absorbed dose to most organs.^12,16^ Fourth, combining effect size measures based on general population (SIR and SMR) and patients treated for hyperthyroidism with surgery or drugs (HR and RR) may not be appropriate, considering that patients with hyperthyroidism may be more or less likely to develop or die from certain cancers than the general population. However, our analysis by internal and external comparison showed similar results, and combining different effect size measures is possible using meta-analysis methods.^37^ Despite these limitations, we believe the results of this meta-analysis provide quantitative information to understand the cancer risk associated with RAI therapy for hyperthyroidism.

## Conclusions

The overall pooled cancer risk after exposure to RAI therapy vs nonexposure was not significant, whereas a linear dose-response association between RAI therapy and solid cancer mortality was observed. These findings suggest that radiation-induced cancer risks following RAI therapy for hyperthyroidism are small and, in observational studies, may only be detectable at higher levels of administered dose. Further research is needed to precisely quantify cancer risks for exposure vs nonexposure to RAI at the level of administered activity currently used in the treatment of hyperthyroidism.

## Notes

Supplement. **eFigure 1.** Overall Malignant Neoplasm Estimates Using Internal and External Comparisons After Radioactive Iodine Treatment for Hyperthyroidism **eFigure 2.** Funnel Plot for Publication Bias **eFigure 3.** Suggested Algorithm for Reaching Risk-of-Bias Judgements and Overall Quality Assessment **eFigure 4.** Forest Plot for Incidence Ratio by Cancer Site **eFigure 5.** Forest Plot for Mortality Ratio by Cancer Site **eTable 1.** Search Queries **eTable 2.** Quality Assessment by Specific Domain of the Included Radiation Epidemiology Studies
