# Supplementary material for: Cancer Risk After Radioactive Iodine Treatment for Hyperthyroidism: A Systematic Review and Meta-analysis
Source: JAMA Netw Open. 2021 Sep 17;4(9):e2125072. doi: 10.1001/jamanetworkopen.2021.25072 (PMC8449277; doi:10.1001/jamanetworkopen.2021.25072)
Supplement: Supplement. — eFigure 1. Overall Malignant Neoplasm Estimates Using Internal and External Comparisons After Radioactive Iodine Treatment for Hyperthyroidism eFigure 2. Funnel Plot for Publication Bias eFigure 3. Suggested Algorithm for Reaching Risk-of-Bias Judgements and Overall Quality Assessment eFigure 4. Forest Plot for Incidence Ratio by Cancer Site eFigure 5. Forest Plot for Mortality Ratio by Cancer Site eTable 1. Search Queries eTable 2. Quality Assessment by Specific Domain of the Included Radiation Epidemiology Studies [file jamanetwopen-e2125072-s001.pdf]

Supplemental Online Content

Shim SR, Kitahara CM, Cha ES, Kim SJ, Bang YJ, Lee WJ. Cancer risk after radioactive iodine treatment for hyperthyroidism: a systematic review and meta-analysis. *JAMA Netw Open.* 2021;4(9):e2125072. doi:10.1001/jamanetworkopen.2021.25072

eFigure 1. Overall Malignant Neoplasm Estimates Using Internal and External Comparisons After Radioactive Iodine Treatment for Hyperthyroidism

eFigure 2. Funnel Plot for Publication Bias

eFigure 3. Suggested Algorithm for Reaching Risk-of-Bias Judgements and Overall Quality Assessment

eFigure 4. Forest Plot for Incidence Ratio by Cancer Site

eFigure 5. Forest Plot for Mortality Ratio by Cancer Site

eTable 1. Search Queries

eTable 2. Quality Assessment by Specific Domain of the Included Radiation Epidemiology Studies

This supplemental material has been provided by the authors to give readers additional information about their work.

© 2021 Shim SR et al. *JAMA Network Open.*

eFigure 1. Overall Malignant Neoplasm Estimates Using Internal and External Comparisons After Radioactive Iodine Treatment for Hyperthyroidism

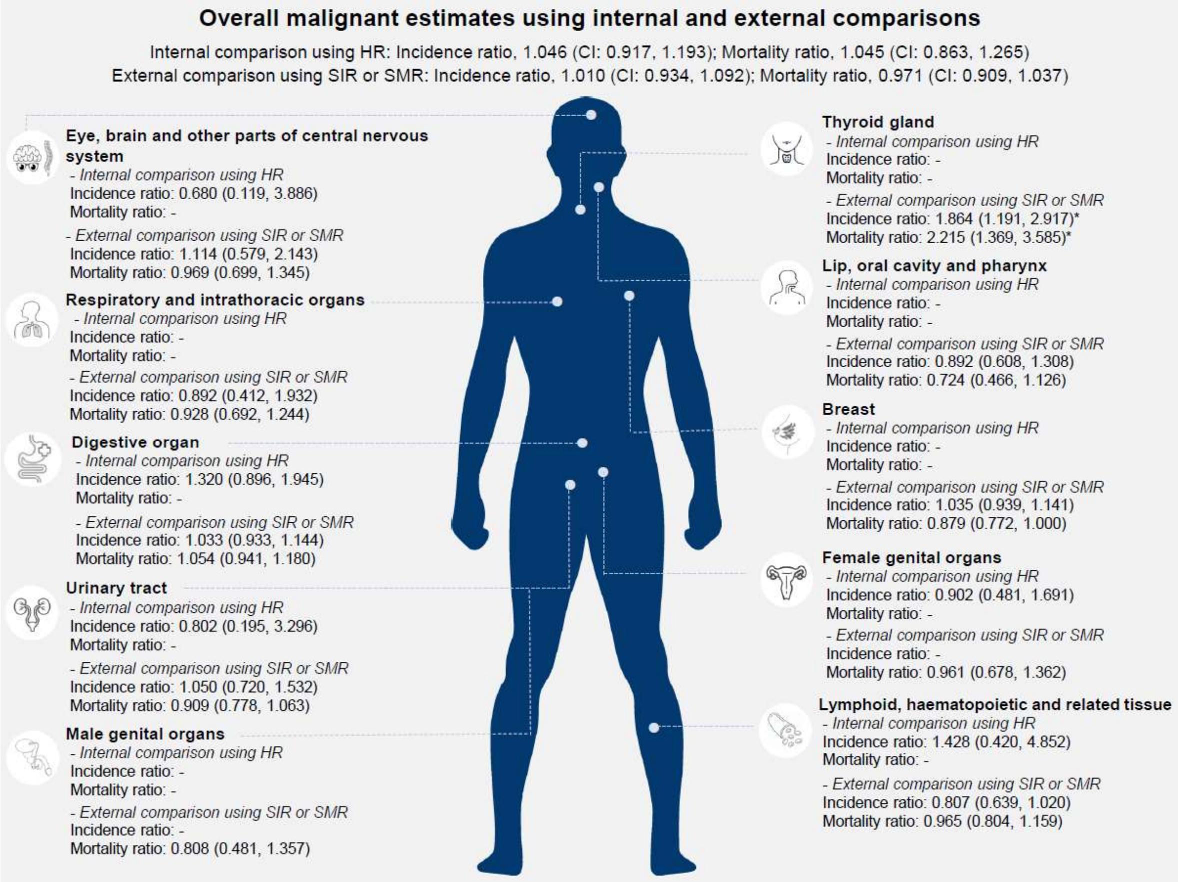

© 2021 Shim SR et al. *JAMA Network Open.*

eFigure 2. Funnel Plot for Publication Bias

A. Incidence Ratio

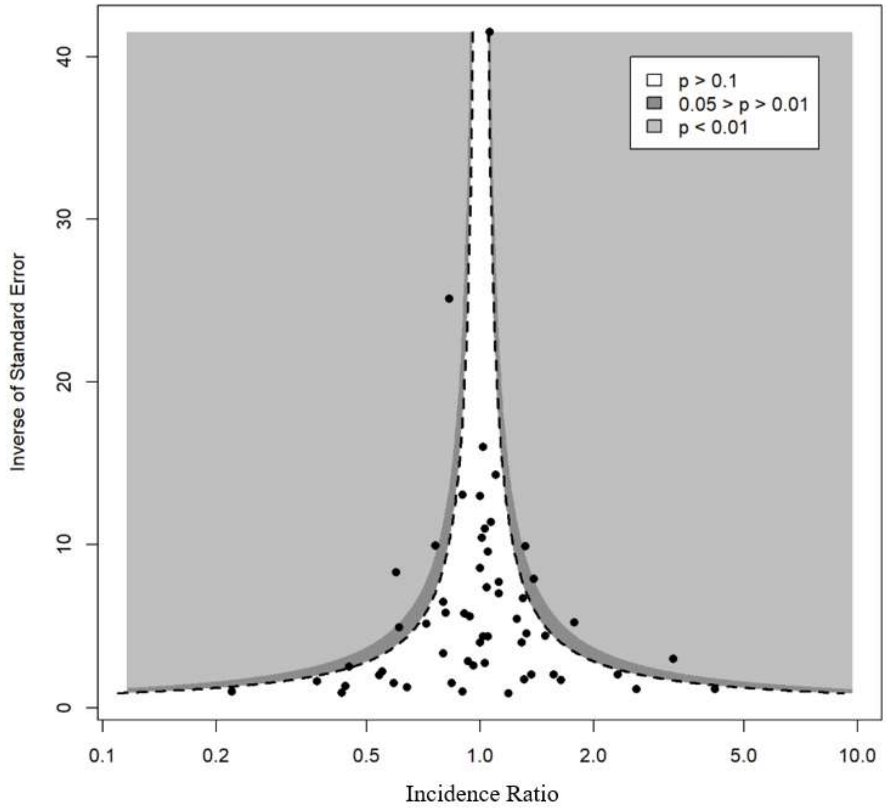

B. Mortality Ratio

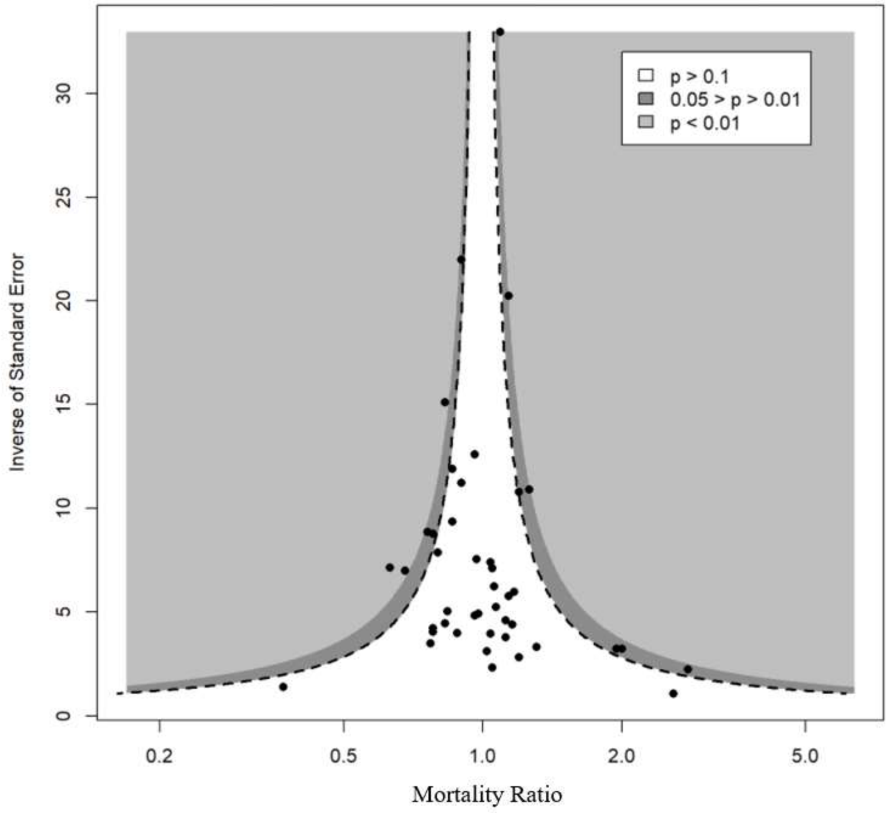

© 2021 Shim SR et al. *JAMA Network Open.*

eFigure 3. Suggested Algorithm for Reaching Risk-of-Bias Judgements and Overall Quality Assessment

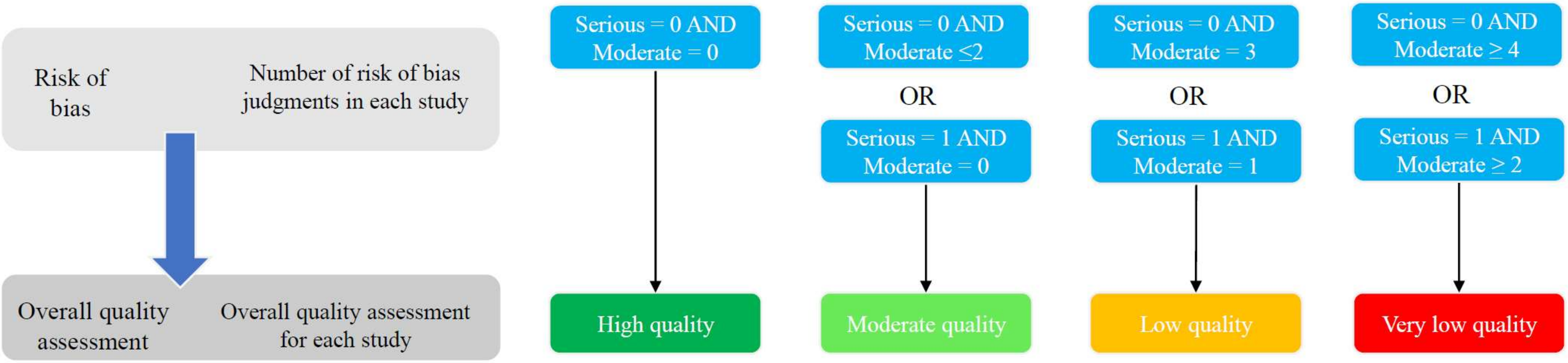

Risk of bias judgement, and overall quality assessment follow the recommendations of United Nations Scientific Committee on the Effects of Atomic Radiation, UNSCEAR 2017.

Risk of bias judgement (low, moderate, serious or critical risk of bias, or no information).

Overall quality assessment (High, Moderate, Low or Very low quality).

© 2021 Shim SR et al. *JAMA Network Open.*

eFigure 4. Forest Plot for Incidence Ratio by Cancer Site

Study or Subgroup	Cancer site	Site sub	Total patients	OR	95% CI
All malignant					
Goldman 1988	All malignant		1762	0.800	[0.591; 1.083]
Holm 1991	All malignant		10207	1.060	[1.011; 1.111]
Ryodi 2015	All malignant		1814	1.030	[0.861; 1.232]
Franklyn 1999	All malignant		7417	0.830	[0.768; 0.897]
Gronich 2020	All malignant		16637	1.010	[0.837; 1.219]
Total (95% CI)				0.951	[0.825; 1.095]
Heterogeneity: $Tau^2 = 0.0194$; $Chi^2 = 29.79$, df = 4 ($P < 0.01$); $I^2 = 87\%$					
Breast					
Goldman 1988	Breast		1762	1.000	[0.612; 1.633]
Holm 1991	Breast		10207	1.020	[0.902; 1.153]
Franklyn 1999	Breast		7417	1.070	[0.901; 1.271]
Gronich 2020	Breast		16637	1.250	[0.871; 1.793]
Total (95% CI)				1.049	[0.954; 1.152]
Heterogeneity: $Tau^2 = 0$; $Chi^2 = 1.2$, df = 3 ($P = 0.75$); $I^2 = 0\%$					
Digestive organ					
Goldman 1988	Digestive organ		1762	0.800	[0.444; 1.442]
Holm 1991	Digestive organ	Stomach	10207	1.050	[0.856; 1.288]
Holm 1991	Digestive organ	Liver	10207	1.330	[0.864; 2.047]
Holm 1991	Digestive organ	Pancreas	10207	1.120	[0.869; 1.444]
Holm 1991	Digestive organ	Intestinecolonrectum	10207	1.100	[0.959; 1.262]
Franklyn 1999	Digestive organ		7417	0.900	[0.774; 1.046]
Gronich 2020	Digestive organ	Intestinecolonrectum	16637	1.490	[0.953; 2.328]
Gronich 2020	Digestive organ	Stomach	16637	0.440	[0.100; 1.933]
Gronich 2020	Digestive organ	Pancreas	16637	0.430	[0.052; 3.577]
Gronich 2020	Digestive organ	Liver and bile ducts	16637	1.640	[0.520; 5.171]
Gronich 2020	Digestive organ	Esophagus	16637	1.190	[0.130; 10.886]
Total (95% CI)				1.049	[0.950; 1.158]
Heterogeneity: $Tau^2 = 0.0037$; $Chi^2 = 11.62$, df = 10 ($P = 0.31$); $I^2 = 14\%$					
Eye, brain and other parts of central nervous system					
Goldman 1988	Eye, brain and other parts of central nervous system	Brain	1762	2.600	[0.462; 14.631]
Holm 1991	Eye, brain and other parts of central nervous system	Brain	10207	1.300	[0.971; 1.740]
Franklyn 1999	Eye, brain and other parts of central nervous system	Brain	7417	0.540	[0.201; 1.449]
Gronich 2020	Eye, brain and other parts of central nervous system	CNS	16637	1.370	[0.521; 3.605]
Gronich 2020	Eye, brain and other parts of central nervous system	Head and neck	16637	0.220	[0.030; 1.607]
Total (95% CI)				1.050	[0.616; 1.789]
Heterogeneity: $Tau^2 = 0.1350$; $Chi^2 = 6.44$, df = 4 ($P = 0.17$); $I^2 = 38\%$					
Female genital organs					
Holm 1991	Female genital organs		10207	1.000	[0.860; 1.163]
Gronich 2020	Female genital organs	Uterus and cervix	16637	1.030	[0.501; 2.116]
Gronich 2020	Female genital organs	Ovary	16637	0.590	[0.163; 2.137]
Total (95% CI)				0.994	[0.858; 1.152]
Heterogeneity: $Tau^2 = 0$; $Chi^2 = 0.65$, df = 2 ($P = 0.72$); $I^2 = 0\%$					
Lip, oral cavity and pharynx					
Holm 1991	Lip, oral cavity and pharynx	Oral cavity	10207	1.020	[0.650; 1.600]
Holm 1991	Lip, oral cavity and pharynx	Salivary glands	10207	0.840	[0.229; 3.086]
Franklyn 1999	Lip, oral cavity and pharynx		7417	0.550	[0.229; 1.323]
Total (95% CI)				0.892	[0.608; 1.308]
Heterogeneity: $Tau^2 = 0$; $Chi^2 = 1.52$, df = 2 ($P = 0.47$); $I^2 = 0\%$					
Lymphoid, haematopoietic and related tissue					
Holm 1991	Lymphoid, haematopoietic and related tissue	Lymphoma	10207	0.720	[0.492; 1.055]
Holm 1991	Lymphoid, haematopoietic and related tissue	Mulitple myeloma	10207	1.050	[0.669; 1.647]
Holm 1991	Lymphoid, haematopoietic and related tissue	Leukemia	10207	0.940	[0.662; 1.334]
Franklyn 1999	Lymphoid, haematopoietic and related tissue		7417	0.610	[0.409; 0.909]
Gronich 2020	Lymphoid, haematopoietic and related tissue	NHL	16637	2.320	[0.879; 6.123]
Gronich 2020	Lymphoid, haematopoietic and related tissue	Leukemia	16637	0.640	[0.131; 3.120]
Total (95% CI)				0.862	[0.655; 1.133]
Heterogeneity: $Tau^2 = 0.0461$; $Chi^2 = 8.76$, df = 5 ($P = 0.12$); $I^2 = 43\%$					
Male genital organs					
Holm 1991	Male genital organs		10207	1.000	[0.795; 1.258]
Gronich 2020	Male genital organs	Prostate	16637	0.960	[0.450; 2.049]
Total (95% CI)				0.997	[0.800; 1.241]
Heterogeneity: $Tau^2 = 0$; $Chi^2 = 0.01$, df = 1 ($P = 0.92$); $I^2 = 0\%$					
Melanoma and other malignant neoplasms of skin					
Gronich 2020	Melanoma and other malignant neoplasms of skin	Melanoma	16637	1.310	[0.427; 4.020]
Total (95% CI)				1.310	[0.427; 4.020]
Heterogeneity: not applicable					
Respiratory and intrathoracic organs					
Holm 1991	Respiratory and intrathoracic organs	Lung	10207	1.320	[1.083; 1.609]
Franklyn 1999	Respiratory and intrathoracic organs		7417	0.600	[0.474; 0.760]
Gronich 2020	Respiratory and intrathoracic organs	Lung	16637	0.930	[0.467; 1.850]
Total (95% CI)				0.902	[0.490; 1.662]
Heterogeneity: $Tau^2 = 0.2499$; $Chi^2 = 25.13$, df = 2 ($P < 0.01$); $I^2 = 92\%$					
Thyroid and other endocrine glands					
Holm 1991	Thyroid and other endocrine glands	Thyroid gland	10207	1.290	[0.789; 2.108]
Holm 1991	Thyroid and other endocrine glands	Parathyroid gland	10207	1.780	[1.223; 2.590]
Franklyn 1999	Thyroid and other endocrine glands	Thyroid gland	7417	3.250	[1.690; 6.250]
Gronich 2020	Thyroid and other endocrine glands	Thyroid gland	16637	0.450	[0.207; 0.977]
Total (95% CI)				1.404	[0.750; 2.629]
Heterogeneity: $Tau^2 = 0.3231$; $Chi^2 = 15.76$, df = 3 ($P < 0.01$); $I^2 = 81\%$					
Urinary tract					
Holm 1991	Urinary tract	Kidney	10207	1.390	[1.084; 1.783]
Holm 1991	Urinary tract	Bladder	10207	1.120	[0.847; 1.482]
Franklyn 1999	Urinary tract		7417	0.760	[0.624; 0.926]
Gronich 2020	Urinary tract	Urinary	16637	0.370	[0.109; 1.252]
Gronich 2020	Urinary tract	Kidney	16637	1.570	[0.603; 4.089]
Total (95% CI)				1.019	[0.717; 1.448]
Heterogeneity: $Tau^2 = 0.0995$; $Chi^2 = 18.18$, df = 4 ($P < 0.01$); $I^2 = 78\%$					
**Total (95% CI)**				**1.015**	**[0.948; 1.088]**
Heterogeneity: $Tau^2 = 0.0233$; $Chi^2 = 138.23$, df = 51 ($P < 0.01$); $I^2 = 63\%$					
Residual heterogeneity: Tau^2 = NA; $Chi^2 = 119.05$, df = 40 ($P < 0.01$); $I^2 = 66\%$					

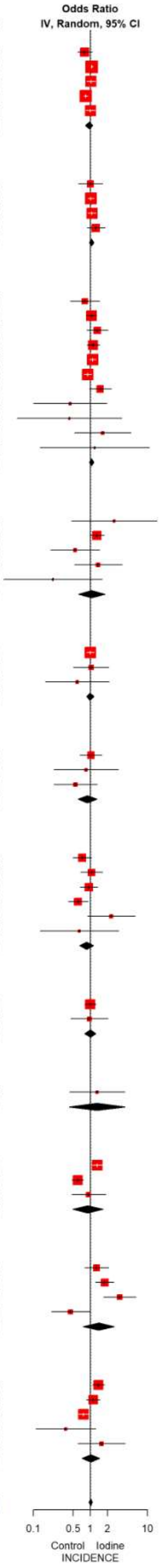

© 2021 Shim SR et al. *JAMA Network Open.*

eFigure 5. Forest Plot for Mortality Ratio by Cancer Site

Study or Subgroup	Cancer site	Site sub	Total patients	OR	95% CI
Lip, oral cavity and pharynx					
Kitahara 2020	Lip, oral cavity and pharynx	Oral cavity	8054	0.780	[0.490; 1.242]
Franklyn 1999	Lip, oral cavity and pharynx		7417	0.370	[0.091; 1.500]
Total (95% CI)				0.724	[0.466; 1.126]
Heterogeneity: $Tau^2 = 0$; $Chi^2 = 0.98$, df = 1 (P = 0.32); $I^2 = 0\%$					
Digestive organ					
Kitahara 2020	Digestive organ	Esophagus	8054	1.310	[0.726; 2.363]
Kitahara 2020	Digestive organ	Stomach	8054	1.140	[0.811; 1.602]
Kitahara 2020	Digestive organ	Colon	8054	0.900	[0.756; 1.072]
Kitahara 2020	Digestive organ	Rectum	8054	1.120	[0.732; 1.713]
Kitahara 2020	Digestive organ	Liver	8054	2.000	[1.092; 3.662]
Kitahara 2020	Digestive organ	Pancreas	8054	0.970	[0.748; 1.258]
Hall 1992	Digestive organ		10552	1.140	[1.035; 1.256]
Franklyn 1999	Digestive organ		7417	0.960	[0.821; 1.122]
Total (95% CI)				1.054	[0.941; 1.180]
Heterogeneity: $Tau^2 = 0.0097$; $Chi^2 = 12.49$, df = 7 (P = 0.09); $I^2 = 44\%$					
Respiratory and intrathoracic organs					
Kitahara 2020	Respiratory and intrathoracic organs	Lung/bronchus	8054	0.830	[0.729; 0.945]
Hall 1992	Respiratory and intrathoracic organs		10552	1.260	[1.053; 1.508]
Franklyn 1999	Respiratory and intrathoracic organs		7417	0.760	[0.609; 0.948]
Total (95% CI)				0.928	[0.692; 1.244]
Heterogeneity: $Tau^2 = 0.0589$; $Chi^2 = 17.02$, df = 2 (P < 0.01); $I^2 = 88\%$					
Breast					
Kitahara 2020	Breast		8054	0.860	[0.700; 1.057]
Hall 1992	Breast		10552	0.860	[0.686; 1.077]
Franklyn 1999	Breast		7417	0.930	[0.728; 1.188]
Total (95% CI)				0.879	[0.772; 1.000]
Heterogeneity: $Tau^2 = 0$; $Chi^2 = 0.28$, df = 2 (P = 0.87); $I^2 = 0\%$					
Female genital organs					
Kitahara 2020	Female genital organs	Uterus	8054	0.680	[0.514; 0.900]
Kitahara 2020	Female genital organs	Ovary	8054	1.060	[0.774; 1.452]
Hall 1992	Female genital organs		10552	1.200	[1.000; 1.439]
Total (95% CI)				0.961	[0.678; 1.362]
Heterogeneity: $Tau^2 = 0.0774$; $Chi^2 = 11.18$, df = 2 (P < 0.01); $I^2 = 82\%$					
Male genital organs					
Kitahara 2020	Male genital organs	Prostate	8054	0.630	[0.479; 0.829]
Hall 1992	Male genital organs		10552	1.070	[0.736; 1.555]
Total (95% CI)				0.808	[0.481; 1.357]
Heterogeneity: $Tau^2 = 0.1123$; $Chi^2 = 5.01$, df = 1 (P = 0.03); $I^2 = 80\%$					
Urinary tract					
Kitahara 2020	Urinary tract	Bladder	8054	0.960	[0.640; 1.439]
Kitahara 2020	Urinary tract	Kidney	8054	0.830	[0.535; 1.289]
Hall 1992	Urinary tract	Kidney	10552	1.170	[0.843; 1.624]
Hall 1992	Urinary tract	Bladder	10552	0.880	[0.538; 1.439]
Franklyn 1999	Urinary tract		7417	0.800	[0.624; 1.026]
Total (95% CI)				0.909	[0.778; 1.063]
Heterogeneity: $Tau^2 = 0$; $Chi^2 = 3.53$, df = 4 (P = 0.47); $I^2 = 0\%$					
Eye, brain and other parts of central nervous system					
Kitahara 2020	Eye, brain and other parts of central nervous system	Brain/CNS	8054	1.040	[0.634; 1.706]
Hall 1992	Eye, brain and other parts of central nervous system	Nervous system	10552	0.770	[0.439; 1.349]
Franklyn 1999	Eye, brain and other parts of central nervous system	Brain	7417	1.200	[0.600; 2.400]
Total (95% CI)				0.969	[0.699; 1.345]
Heterogeneity: $Tau^2 = 0$; $Chi^2 = 1.09$, df = 2 (P = 0.58); $I^2 = 0\%$					
Thyroid and other endocrine glands					
Kitahara 2020	Thyroid and other endocrine glands	Thyroid gland	8054	2.590	[0.422; 15.905]
Hall 1992	Thyroid and other endocrine glands	Thyroid gland	10552	1.950	[1.061; 3.583]
Franklyn 1999	Thyroid and other endocrine glands	Thyroid gland	7417	2.780	[1.159; 6.666]
Total (95% CI)				2.215	[1.369; 3.585]
Heterogeneity: $Tau^2 = 0$; $Chi^2 = 0.46$, df = 2 (P = 0.80); $I^2 = 0\%$					
Lymphoid, haematopoietic and related tissue					
Ron 1998	Lymphoid, haematopoietic and related tissue	CLL	7474	1.050	[0.453; 2.433]
Ron 1998	Lymphoid, haematopoietic and related tissue	Non-CLL	7474	1.120	[0.668; 1.879]
Ron 1998	Lymphoid, haematopoietic and related tissue	Lymphoma	7474	1.160	[0.743; 1.812]
Ron 1998	Lymphoid, haematopoietic and related tissue	Myeloma	7474	1.020	[0.542; 1.919]
Hall 1992	Lymphoid, haematopoietic and related tissue	Lymphoma	10552	0.780	[0.481; 1.264]
Hall 1992	Lymphoid, haematopoietic and related tissue	Leukemia	10552	0.980	[0.658; 1.460]
Franklyn 1999	Lymphoid, haematopoietic and related tissue		7417	0.840	[0.570; 1.239]
Total (95% CI)				0.965	[0.804; 1.159]
Heterogeneity: $Tau^2 = 0$; $Chi^2 = 2.28$, df = 6 (P = 0.89); $I^2 = 0\%$					
All malignant					
Hall 1992	All malignant		10552	1.090	[1.027; 1.157]
Ryodi 2015	All malignant		1814	1.050	[0.797; 1.384]
Franklyn 1999	All malignant		7417	0.900	[0.823; 0.984]
Giesecke 2018	All malignant		10992	1.040	[0.798; 1.356]
Total (95% CI)				1.009	[0.885; 1.151]
Heterogeneity: $Tau^2 = 0.0113$; $Chi^2 = 12.31$, df = 3 (P < 0.01); $I^2 = 76\%$					
**Total (95% CI)**				**0.975**	**[0.915; 1.037]**
Heterogeneity: $Tau^2 = 0.0169$; $Chi^2 = 98.07$, df = 42 (P < 0.01); $I^2 = 57\%$					

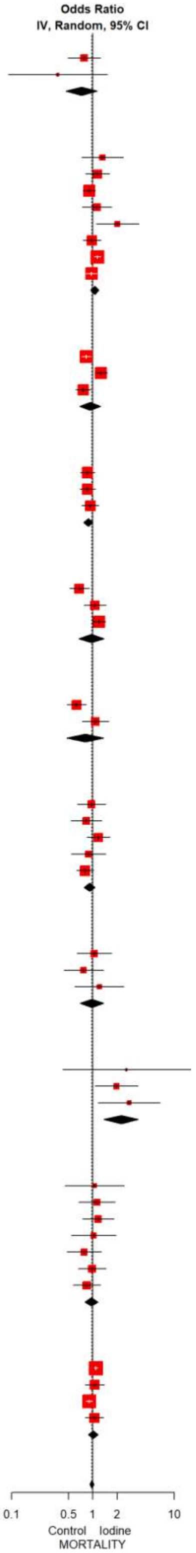

© 2021 Shim SR et al. *JAMA Network Open.*

eTable 1. Search Queries

**PubMed and Cochrane Library**		
		1. Disease; benign thyroid disease<br>("Hyperthyroidism"[Mesh] OR "Hyperthyroidism"[tiab] OR "Graves disease"[tiab] OR "Graves diseases"[tiab] OR "Exophthalmic Goiter"[tiab] OR "Thyrotoxicosis"[Mesh] OR "Thyrotoxicosis"[tiab])
		2. Intervention<br>("Iodine"[Mesh] OR "Iodine"[tiab] OR "Iodine-131" [Supplementary Concept] OR "iodine-131 anti-B1 antibody" [Supplementary Concept] OR "Iodine 131"[tiab] OR "Iodine-131"[tiab] OR "Iodine Radioisotopes"[Mesh] OR "Iodine Radioisotopes"[tiab] OR "Radioactive iodine"[tiab])
		3. Outcome<br>("Neoplasms"[Mesh] OR "Neoplasm"[tiab] OR "Neoplasms"[tiab] OR "Cancer"[tiab] OR "cancers"[tiab] OR "Tumors"[tiab] OR "Tumor"[tiab] OR "carcinoma"[tiab] OR "carcinomas"[tiab])
**Embase**		
		1. Disease; benign thyroid disease<br>('thyrotoxicosis'/exp OR 'hyperthyroidism'/exp OR 'Graves Disease':ab,ti OR Basedow:ab,ti OR 'Exophthalmic Goiter':ab,ti OR 'Exophthalmic Goiters':ab,ti OR hyperthyroidism:ab,ti OR thyrotoxicosis:ab,ti)
		2. Intervention<br>('iodine'/exp OR 'radioactive iodine'/exp OR 'iodine radioisotopes':ab,ti OR 'radioactive iodine':ab,ti OR radioiodine:ab,ti OR 'radio-iodine':ab,ti OR 'iodine-131':ab,ti OR 'iodine 131':ab,ti)
		3. Outcome<br>('neoplasm'/exp OR Cancer:ab,ti OR cancers:ab,ti OR Neoplasm:ab,ti OR Neoplasms:ab,ti OR Tumors:ab,ti OR Tumor:ab,ti OR Tumours:ab,ti OR Tumour:ab,ti OR carcinoma:ab,ti OR carcinomas:ab,ti) AND 'human'/de AND 'article'/it

© 2021 Shim SR et al. *JAMA Network Open.*

eTable 2. Quality Assessment by Specific Domain of the Included Radiation Epidemiology Studies

Study	Overall quality assessment	Risk of bias domain	Risk of bias judgement	Description
Goldman, 1988[28]	Moderate	Study participants (selection bias)	Low	A cohort study of US or Massachusetts standard population. The study population was all women with a diagnosis of hyperthyroidism who had been treated at the Massachusetts General Hospital Thyroid Unit between January 1, 1946 and December 21, 1964. The control was US or Massachusetts standard population, and the analyzed sample size was 1,762 (RAI only 607, RAI & other 799, no RAI 356).
		Exposure (performance bias)	Moderate	There was no adequate explanation for the dose estimation procedure especially for individual organs. However, SIR was shown by dose level of administered activity.
		Outcomes (detection bias)	Low	There was an objective measure of outcome (SIR and SMR) which was appropriately justified and selected. No any systematic outcome measurement errors.
		Design-specific bias (attrition bias, other biases)	Low	The period of follow-up was reasonably complete (treatment period, 1946-1964 and follow-up 17.2 years). Lag time 1 year. The follow-up began one year after the date of her primary treatment for hyperthyroidism at Massachusetts General Hospital.
		Confounder control (other biases)	Moderate	The essential covariates (age, calendar time, sex, race-specific, region) have been adjusted. The type of hyperthyroidism (Graves' disease and Toxic nodular goiter) has not been considered.
		Statistical methods (other biases)	Low	The statistical methods were appropriate for the available data (USDR computer program and Cox PH model used for SIR & SMR calculating). They provided additional sensitivity analysis results (by age at treatment and year of treatment).
		Reporting (other biases)	Low	The reported results were complete and unbiased (stratified reporting by severity of hyperthyroidism, all organ sites, treatment methods, and dose level of administered activity).
		Conflict of interest	NI	No information
Holm, 1991[8]	Moderate	Study participants (selection bias)	Low	A Swedish 7 hospital cohort of Swedish Cancer Register population. An analyzed sample size was 10,207 patients (82% women and 18% men) under the age of 75 were identified as having received RAI therapy for hyperthyroidism between 1950 and 1975. The control was Swedish Cancer Register population.
		Exposure (performance bias)	Moderate	The ICRP and data from Edmonds and Smith were used to estimate the radiation dose from RAI treatment to various organs. However, SIR was not shown by dose level of administered activity.
		Outcomes (detection bias)	Low	There was an objective measure of outcome (SIR) which was appropriately justified and selected. No any systematic outcome measurement errors.
		Design-specific bias (attrition bias, other biases)	Low	The follow-up was reasonably complete (treatment period, 1950-1975 and follow-up 15 years). Lag time 1 and 10 years. All patients were considered to be at risk from 1 year after the initial RAI treatment or from 1958 if first treatment occurred prior to that year.

© 2021 Shim SR et al. *JAMA Network Open.*

		Confounder control (other biases)	Low	The essential covariates (age, sex, calendar year, region, dose) have been adjusted. The type of hyperthyroidism (Graves' disease and Toxic nodular goiter) has been considered.
		Statistical methods (other biases)	Moderate	The statistical methods were appropriate for the available data (Poisson distribution assumption and chi-suqare test). They did not provide additional sensitivity analysis results and dose-response analysis.
		Reporting (other biases)	Low	The reported results were complete and unbiased (stratified reporting by hyperthyroidism types, all organ sites, follow-up period 1 & 10 years).
		Conflict of interest	NI	No information
Hall, 1992[9]	Moderate	Study participants (selection bias)	Low	A Swedish 7 hospital cohort of Swedish Cancer Register population. An analyzed sample size was 10,552 (93% for hyperthyroidism, 7% for non-specified thyroid disease) under the age of 75 were identified as having received RAI therapy for hyperthyroidism between 1950 and 1975. The control was Swedish Cancer Register population.
		Exposure (performance bias)	Low	The ICRP tables, the mean 24-hr uptake, and the mean administered activity of RAI were used to calculate the mean radiation dose to various organs. SMR was shown by dose level of administered activity.
		Outcomes (detection bias)	Low	There was an objective measure of outcome (SMR) which was appropriately justified and selected. No any systematic outcome measurement errors.
		Design-specific bias (attrition bias, other biases)	Low	The follow-up was resonably complete (treatment period, 1950-1975 and follow-up 15 years). Lag time 1 and 10 years. All patients were considered to be at risk from the initial RAI treatment until death or December 31, 1986
		Confounder control (other biases)	Low	The essential covariates (age, sex, calendar year, region, dose) have been adjusted. The type of hyperthyroidism (Graves' disease and Toxic nodular goiter) has been considered.
		Statistical methods (other biases)	Moderate	The statistical methods were appropriate for the available data (Poisson distribution assumption and chi-square test). They did not provide additional sensitivity analysis results and dose-response analysis.
		Reporting (other biases)	Low	The reported results were complete and unbiased (stratified reporting by all organ sites, age groups, follow-up period, and dose level of administered activity).
		Conflict of interest	NI	No information
Ron, 1998[10]	High	Study participants (selection bias)	Low	A TTFUS cohort study of US standard population. The study population was a patient with hyperthyroidism treated between January 1, 1946 and December 21, 1964 at one of 26 study clinics. The control was US standard population, and the analyzed sample size was 35,593 patients (RAI only 8054, RAI & other 20949, Surgery with or without drugs 10876, Drugs only 1177).
		Exposure (performance bias)	Low	Doses from RAI to 17 organs were estimated for study subjects by multiplying the amount of administered activity by the dose factors (age and 24-hour thyroid uptake) provided for each organ in current ICRP tables. RR was shown by dose level of administered activity.
		Outcomes (detection bias)	Low	There was an objective measure of outcome (SMR, RR and ERR) which was appropriately justified and selected. No any systematic outcome measurement errors.

© 2021 Shim SR et al. *JAMA Network Open.*

		Design-specific bias (attrition bias, other biases)	Low	The follow-up was resonably complete (treatment period, 1946-1964 and follow-up 21 years). Lag time 1 to over 10 years. The follow-up was extended until December 31, 1990.
		Confounder control (other biases)	Low	The essential covariates (age, sex, race, calendar year, time since treatment, dose) have been adjusted. The type of hyperthyroidism (Graves' disease and Toxic nodular goiter) has been considered.
		Statistical methods (other biases)	Low	The statistical methods were appropriate for the available data (EPICURE software and Poisson distribution assumption used for SMR, RR, ERR calculating). They provided additional sensitivity analysis results (dose-response analysis).
		Reporting (other biases)	Low	The reported results were complete and unbiased (stratified reporting by all organ sites, study entry year groups, hyperthyroidism types, treatment methods, and dose level of administered activity).
		Conflict of interest	NI	No information
Franklyn, 1999[11]	Very Low	Study participants (selection bias)	Low	A cohort study of UK regional cancer register population. All patients who had been treated for hyperthyroidism with RAI in the West Midlands region of the UK between 1950 and 1991. The control was UK Regional Cancer Register and the analyzed sample size was 7,417 patients.
		Exposure (performance bias)	Serious	There was no adequate explanation for the dose estimation procedure especially for individual organs. SIR and SMR were not shown by dose level of administered activity (the overall incidence of uterine cancer and bladder cancer were only described in main body, not in the regular table).
		Outcomes (detection bias)	Low	There was an objective measure of outcome (SIR and SMR) which was appropriately justified and selected. No any systematic outcome measurement errors.
		Design-specific bias (attrition bias, other biases)	Low	The follow-up was reasonably complete (treatment period, I131 for 1950-1991 & control for 1971-1991, and follow-up 9.7 years). The follow-up was computerized Birmingham Thyroid Follow-up Register.
		Confounder control (other biases)	Moderate	The essential covariates (age, sex, calendar year, period) have been adjusted. The type of hyperthyroidism (Graves' disease and Toxic nodular goiter) has not been considered.
		Statistical methods (other biases)	Moderate	The statistical methods were appropriate for the available data (Poisson regression used for SIR and SM calculating). They did not provide additional sensitivity analysis results and dose-response analysis.
		Reporting (other biases)	Moderate	The reported results were not complete and unbiased (only main outcomes of SIR and SMR were reported without stratified reporting by covariates).
		Conflict of interest	NI	No information
Hahn, 2001[29]	Moderate	Study participants (selection bias)	Low	A cohort study of German democratic Republic's cancer registry population. Exposed and nonexposed patients were recruited from 10 German hospitals that had conducted thyroid examinations in children. Patients were included in the RAI group (exposed group) if records existed for at least one administration of RAI for diagnostic purposes for the age of 18 years. The nonexposed group (German democratic Republic's cancer registry) consisted of patients who had been examined because of suspected thyroid disease. The analyzed sample size was 789 (RAI) and 1118 (non-exposed)

© 2021 Shim SR et al. *JAMA Network Open.*

		Exposure (performance bias)	Low	The thyroid dose from RAI was calculated according to the biokinetic model of ICRP Report No. 53 (21). OR was shown by dose level of administered activity.
		Outcomes (detection bias)	Low	There was an objective measure of outcome (SIR, RR and OR) which was appropriately justified and selected. No any systematic outcome measurement errors.
		Design-specific bias (attrition bias, other biases)	Low	The follow-up was resonably complete (treatment period, I131 for 1958-1978 & non-expose for 1959-1986). Person-time for exposed subjects was defined as the period between the first test using RAI uptake and the study examination; person-time for nonexposed subjects was defined as the period between the initial referral for one of the examinations fulfilling the inclusion criteria for this group and the study examination.
		Confounder control (other biases)	Low	The essential covariates (age, sex, calendar year, time since treatment, dose) have been adjusted. The type of hyperthyroidism (Graves' disease and Toxic nodular goiter) has been considered.
		Statistical methods (other biases)	Moderate	The statistical methods were appropriate for the available data (logistic regression used for SIR, RR, and OR calculating). They did not provide additional sensitivity analysis results.
		Reporting (other biases)	Low	The reported results were complete and unbiased (stratified reporting by study entry year groups, hyperthyroidism types, and dose level of administered activity).
		Conflict of interest	NI	No information
Dickman, 2003[27]	Moderate	Study participants (selection bias)	Low	A Swedish 7 hospital cohort of Swedish Cancer Register population. An analyzed sample size was 24010 (no prior exposure to external radiotherapy) under the age of 75 were identified as having received RAI therapy for hyperthyroidism between 1952 and 1969. The control was Swedish Cancer Register population.
		Exposure (performance bias)	Low	The ICRP tables, the mean 24-hr uptake, and the mean administered activity of RAI were used to calculate the mean radiation dose to various organs. SIR was shown by absorbed thyroid dose.
		Outcomes (detection bias)	Low	There was an objective measure of outcome (SIR) which was appropriately justified and selected. No any systematic outcome measurement errors.
		Design-specific bias (attrition bias, other biases)	Low	The follow-up period commenced at the date of first RAI administration or 1 January 1958 if the patient was first exposed prior to 1958. Accumulation of person-time at risk began 2 years after the date of first RAI administration. Person-time at risk was accumulated until the date of diagnosis of thyroid cancer, date of death, date of emigration or 31 December 1998, whichever occurred first. Lag time 2 to 20 and over 20 years.
		Confounder control (other biases)	Moderate	The essential covariates (age at first exposure, time since first exposure, absorbed RAI dose to the thyroid gland and gender) have been adjusted. However, the type of hyperthyroidism (Graves' disease and Toxic nodular goiter) has not been considered.
		Statistical methods (other biases)	Moderate	The statistical methods were appropriate for the available data (EPICURE software and Poisson distribution assumption). They did not provide additional sensitivity analysis results.
		Reporting (other biases)	Low	The reported results were complete and unbiased (stratified reporting by time since first exposure, absorbed RAI dose to the thyroid gland and gender).

© 2021 Shim SR et al. *JAMA Network Open.*

		Conflict of interest	NI	No information
Metso, 2007[30]	Moderate	Study participants (selection bias)	Low	A cohort study of Finland national wide Hospital Discharge Registry population. The study population was a patient with hyperthyroidism treated between January 1965 and June 2002 at Tampere University Hospital. Choosing an age- and gender-matched control subject for each patient from the Population Register Centre formed a reference group. The analyzed sample size was 1,399 of RAI and 1,465 of thyroidectomy.
		Exposure (performance bias)	Moderate	There was no adequate explanation for the dose estimation procedure especially for individual organs. However, RR was shown by dose level of administered activity.
		Outcomes (detection bias)	Low	There was an objective measure of outcome (RR & HR) which was appropriately justified and selected. No any systematic outcome measurement errors.
		Design-specific bias (attrition bias, other biases)	Low	The follow-up was reasonably complete (treatment period, 1966-2003 and follow-up 9 years (RAI) & 9.4 years (control)). Lag time 3 months. The follow-up period of the control subject started at the same time as that of the corresponding patient. In both patient and control groups, the follow-up ended on the date of death, emigration, or the common closing date (December 2003)
		Confounder control (other biases)	Low	The essential covariates (age, sex, treatment type, etiology of hyperthyroidism, dose) have been adjusted. The type of hyperthyroidism (Graves' disease and Toxic nodular goiter) has been considered.
		Statistical methods (other biases)	Moderate	The statistical methods were appropriate for the available data (Kaplan-Meier analysis with the log-rank test used for RR and HR calculating). They did not provide additional sensitivity analysis results.
		Reporting (other biases)	Low	The reported results were complete and unbiased (stratified reporting by all organ sites, age at first treatment, etiology of hyperthyroidism, infectious diseases, endocrine diseases, cardiovascular diseases, and dose level of administered activity).
		Conflict of interest	Low	They provided a statement of conflict of interest to all contributors.
Ryodi, 2015[31]	Low	Study participants (selection bias)	Low	A cohort study of Finland national wide Hospital Discharge Registry population. The study population was a patient with hyperthyroidism treated between January 1986 and December 2007 in Finland from the nationwide Hospital Discharge Registry maintained by the National Institute for Health and Welfare. The reference population was formed by randomly choosing three age- and sex-matched control subjects for each patient from the comprehensive national population register. The analyzed sample size was 1,814 (1485 women and 329 men).
		Exposure (performance bias)	Serious	There was no adequate explanation for the dose estimation procedure especially for individual organs. RR and HR were not shown by dose level of administered activity.
		Outcomes (detection bias)	Low	There was an objective measure of outcome (RR & HR) which was appropriately justified and selected. No any systematic outcome measurement errors.
		Design-specific bias (attrition bias, other biases)	Low	The follow-up was resonably complete (treatment period, 1986-2007 and follow-up 10 years). Lag time 3 months. The follow-up of the patients for cancer incidence started 3 months after the treatment, ie, thyroidectomy or the first dose of RAI, and on the same day for the corresponding controls.

© 2021 Shim SR et al. *JAMA Network Open.*

		Confounder control (other biases)	Low	The essential covariates (age, sex, treatment type, etiology of hyperthyroidism) have been adjusted. The type of hyperthyroidism (Graves' disease and Toxic nodular goiter) has been considered.
		Statistical methods (other biases)	Moderate	The statistical methods were appropriate for the available data (Cox regression multivariate analysis used for RR and HR calculating). They did not provide additional sensitivity analysis results and dose-response analysis.
		Reporting (other biases)	Low	The reported results were complete and unbiased (stratified reporting by all organ sites, sex, etiology of hyperthyroidism).
		Conflict of interest	Low	They provided a statement of conflict of interest to all contributors.
Giesecke, 2018[14]	Moderate	Study participants (selection bias)	Low	A Swedish health care register cohort. In this study, patients treated with radioiodine for hyperthyroidism were identified using the Stockholm Radioiodine Cohort, a database compiled from the hospital records of Radiumhemmet at Karolinska University Hospital. Although the database is part of a nationwide equivalent that covers the years 1950–2000, it is considered to be essentially complete for the greater Stockholm area only during the years 1976–2000. An analyzed sample size was 10,992 (10250 for RAI), 742 for Thyroidectomy). The control was thyroidectomy and Swedish Cancer Register population.
		Exposure (performance bias)	Serious	There was no adequate explanation for the dose estimation procedure especially for individual organs. HR was not shown by dose level of administered activity.
		Outcomes (detection bias)	Low	There was an objective measure of outcome (HR) which was appropriately justified and selected. No any systematic outcome measurement errors.
		Design-specific bias (attrition bias, other biases)	Low	The follow-up was reasonably complete (treatment period, 1976-2000 and follow-up 16.3-22.3 years). The Patient Register is managed by the Swedish National Board of Health and Welfare, and has been validated and used for research previously. The study cohort was compiled by merging extracted data on Stockholm residents for the years 1976–2000. All individuals (both radioiodine treated and surgically treated) were then matched to the entire Patient Register for the period 1969–2000 to find other diagnoses, related to any earlier hospitalizations, that would constitute relevant baseline co-morbidity.
		Confounder control (other biases)	Low	The essential covariates (age, sex, smoking history, BMI, Clalit district, socioeconomic status, diabetes mellitus, hypertension, pharmacy of aspirin and of statins, and adherence to mammography) have been adjusted. The type of hyperthyroidism (Graves' disease and Toxic nodular goiter) has been considered.
		Statistical methods (other biases)	Low	The statistical methods were appropriate for the available data (propensity score matching, inverse probability weighting, and Cox PH regression used for HR calculating). They provided additional sensitivity analysis results (by propensity score matching and inverse probability weighting, and only subjects aged 35–75 years.).
		Reporting (other biases)	Low	The reported results were complete and unbiased (stratified reporting by all-cause mortality, cardiovascular disease, age at treatment, year of treatment, co-morbidities at baseline, hyperthyroidism types, and treatment methods).
		Conflict of interest	Low	They provided a statement of conflict of interest to all contributors.
Gronich, 2020[15]	Low	Study participants (selection bias)	Low	A cohort study of Israel Clalit Health service register population. We carried out a historical cohort study of all patients with a new diagnosis of hyperthyroidism (thyrotoxicosis, thyroid nodular goiter, or benign

© 2021 Shim SR et al. *JAMA Network Open.*

				neoplasm of thyroid), between January 1, 2002 and June 30, 2015 who were treated with any thionamide drug (propylthiouracil or thiamazole) or with RAI. The control was thionamide and Israel Clalit Health service register.
		Exposure (performance bias)	Serious	There was no adequate explanation for the dose estimation procedure especially for individual organs. HR was not shown by dose level of administered activity.
		Outcomes (detection bias)	Low	There was an objective measure of outcome (HR) which was appropriately justified and selected. No any systematic outcome measurement errors.
		Design-specific bias (attrition bias, other biases)	Low	The follow-up was reasonably complete (treatment period, 2002-2015). Date of entry to the cohort was the date of first prescription of a thionamide or radioiodine. If a patient was treated with thionamide and later with radioiodine, time of follow-up starting at beginning of thionamides and before receiving radioiodine was added to the follow-up time of thionamide treatment to prevent immortal time bias. Thus, for this patient, follow-up time for radioiodine was started at the date of administration of radioiodine.
		Confounder control (other biases)	Moderate	The essential covariates (age, sex, smoking history, BMI, Clalit district, socioeconomic status, diabetes mellitus, hypertension, pharmacy of aspirin and of statins, and adherence to mammography) have been considered. The type of hyperthyroidism (Graves' disease and Toxic nodular goiter) has not been considered.
		Statistical methods (other biases)	Low	The statistical methods were appropriate for the available data (propensity score matching, inverse probability weighting, and Cox PH regression used for HR calculating). For sensitivity analysis we stratified the cohort by calendar year at cohort entry to account for difference, if it existed, in radioiodine dose over the years.
		Reporting (other biases)	Low	The reported results were complete and unbiased (stratified reporting by all-cause incidence, NHL only, follow-up, and treatment methods).
		Conflict of interest	Low	They provided a statement of conflict of interest to all contributors.
Kitahara, 2020[16]	High	Study participants (selection bias)	Low	A TTFUS cohort study of US standard population. The study population was a patient with hyperthyroidism treated between January 1, 1946 and December 21, 1964 at one of 26 study clinics. The control was US standard population, and the analyzed sample size was 31,363 patients (RAI only 7474, RAI & other 12115, Surgery only 800, Drugs only 1138, Drugs and surgery 9817)
		Exposure (performance bias)	Low	RAI photon and electron spectra from the ICRP were used to compute the S values (mean absorbed dose in a target region per unit disintegration of RAI in a source region) on the adult reference voxel phantoms adopted by the ICRP for all important combinations of source and target regions. HR was shown by dose level of administered activity.
		Outcomes (detection bias)	Low	There was an objective measure of outcome (SMR and HR) which was appropriately justified and selected. No any systematic outcome measurement errors.
		Design-specific bias (attrition bias, other biases)	Low	The follow-up was reasonably complete (treatment period, 1946-1964 and follow-up 26 years). Lag time 5 years. Follow-up of US patients was recently extended through December 31, 2014.

© 2021 Shim SR et al. *JAMA Network Open.*

		Confounder control (other biases)	Low	The essential covariates (age, sex, birth cohort, other risk factors, dose) have been adjusted. The type of hyperthyroidism (Graves' disease and Toxic nodular goiter) has been considered.
		Statistical methods (other biases)	Low	The statistical methods were appropriate for the available data (EPICURE software and Cox PH model used for SMR, HR calculating). They provided additional sensitivity analysis results (dose-response analysis).
		Reporting (other biases)	Low	The reported results were complete and unbiased (stratified reporting by all organ sites, follow-up, hyperthyroidism types, treatment methods, and dose level of administered activity).
		Conflict of interest	Low	They provided a statement of conflict of interest to all contributors.

TTFUS, the cooperative thyrotoxicosis therapy follow-up study cohort at 25 US and 1 UK hospitals. Sweden, Swedish cohort of 7 hospitals.

Risk of bias judgment and overall quality assessment follow the recommendations of the United Nations Scientific Committee on the Effects of Atomic Radiation (UNSCEAR 2017)[18].

Risk of bias judgment (order with less bias; low, moderate, serious, critical risk of bias, or no information). Overall quality assessment (order of good quality; high, moderate, low, or very low quality).

In all previous studies in 2000, the conflict of interest (COI) domain was excluded while assessing the overall quality because there was no information about COI.

© 2021 Shim SR et al. *JAMA Network Open.*
